# Decentralized clinical trials for medications to reduce the risk of dementia: Consensus report and guidance

**DOI:** 10.1002/alz.13891

**Published:** 2024-06-02

**Authors:** Leanne Howard, Carla Abdelnour, Erin L. Abner, Ricardo F. Allegri, Hiroko H. Dodge, Serge Gauthier, Camilla M. Hoyos, Gregory A. Jicha, Patrick G. Kehoe, Catherine J. Mummery, Adesola Ogunniyi, Nikolaos Scarmeas, Xiaoying Chen, Jodie R. Titiner, Christopher R. Weber, Clinical Trial Methodology Working review group members, Ruth Peters

**Affiliations:** ^1^ Brain Health The George Institute for Global Health Sydney New South Wales Australia; ^2^ School of Population Health University of New South Wales Sydney New South Wales Australia; ^3^ Department of Neurology and Neurological Sciences Stanford University School of Medicine Stanford USA; ^4^ Department of Epidemiology and Environmental Health University of Kentucky Lexington USA; ^5^ Departament of Cognitive Neurology, Neuropsychology, and Neuropsychiatry Instituto de Investigaciones Neurologicas Fleni Buenos Aires Argentina; ^6^ Department of Neurosciences Universidad de la Costa Barranquilla Colombia; ^7^ Department of Neurology Massachusetts General Hospital and Harvard Medical School Boston USA; ^8^ Department of Neurology and Neurosurgery McGill University Montréal Québec Canada; ^9^ Medicine, Human and Health Sciences Macquarie University Sydney NSW Australia; ^10^ Translational Health Sciences Bristol Medical School University of Bristol Bristol UK; ^11^ Neurodegenerative Diseases University College London London UK; ^12^ Department of Medicine University of Ibadan Ibadan Nigeria; ^13^ 1st Department of Neurology Aiginition Hospital Medical School National and Kapodistrian University of Athens Athens Greece; ^14^ Department of Neurology Columbia University New York New York USA; ^15^ International Society to Advance Alzheimer's Research and Treatment Alzheimer's Association Chicago USA; ^16^ School of Public Health Imperial College London London UK

**Keywords:** decentralized clinical trials, Delphi process, dementia prevention, recommendations, remote clinical trials

## Abstract

**INTRODUCTION:**

Recent growth in the functionality and use of technology has prompted an increased interest in the potential for remote or decentralized clinical trials in dementia. There are many potential benefits associated with decentralized medication trials, but we currently lack specific recommendations for their delivery in the dementia field.

**METHODS:**

A modified Delphi method engaged an expert panel to develop recommendations for the conduct of decentralized medication trials in dementia prevention. A working group of researchers and clinicians with expertise in dementia trials further refined the recommendations.

**RESULTS:**

Overall, the recommendations support the delivery of decentralized trials in dementia prevention provided adequate safety checks and balances are included. A total of 40 recommendations are presented, spanning aspects of decentralized clinical trials, including safety, dispensing, outcome assessment, and data collection.

**DISCUSSION:**

These recommendations provide an accessible, pragmatic guide for the design and conduct of remote medication trials for dementia prevention.

**Highlights:**

- Clinical trials of medication have begun adopting decentralized approaches.
- Researchers in the field lack guidance on what would be appropriate circumstances and frameworks for what would be appropriate circumstances and frameworks for the use of decentralized trial methods in dementia prevention.
- The present report provides consensus‐based expert recommendations for decentralized clinical trials for dementia prevention.

## 1 BACKGROUND

The development of online communication technologies and the increasing acceptance of remote cognitive testing, accelerated by the global pandemic, have provided greater opportunities for delivering remote or decentralized medication trials in dementia.^1^ Decentralized or remote trials involve conducting some or all of the trial elements outside the conventional settings of clinical trial sites.^2^, ^3^, ^4^ There are many potential benefits attached to decentralized clinical trials. Such trials may support greater equity of access making participation accessible to those who are unable to access traditional clinic sites due to geography, mobility, cost, time, or caring constraints.^2^, ^3^, ^4^ This, in turn, may support the inclusion of more representative populations and generate more broadly applicable data. Conversely, there are also risks inherent in remote or decentralized trial delivery that must be carefully monitored and managed. For example, clinical and safety responsibilities that protect participants remain critical despite potential reductions in face‐to‐face contact and access to standard clinical care practices.^5^, ^6^ In order for decentralized trials to succeed, trial medications must be administered appropriately, and validated outcome assessments are needed.^2^, ^3^, ^4^ These considerations may vary in relative importance depending on the type of medication or medications, assessments, and population under study, but remain essential components for any decentralized trial.^2^, ^3^, ^4^ Such trials must also ensure they are able to comply with the regulatory requirements in the region where the trial is conducted. All these considerations may place constraints on the use of online or remote data collection technologies, and the extent to which a fully decentralized model can be achieved is uncertain at present. With an emerging focus on earlier treatment and prevention, and greater attention on the potential for repurposing medications alongside testing novel anti‐Alzheimer's medications directly, the number of medication trials in dementia prevention and treatment are increasing.^7^ Although general recommendations for decentralized trials are now being made available,^2^, ^4^ condition‐specific guidelines for medication trials in dementia are lacking. Targeted recommendations for decentralized remote dementia trials may provide practical guidance for appropriate trial design and delivery specifically for dementia.

The aim of the present study was to generate pragmatic and practical condition‐specific recommendations for the design and delivery of decentralized clinical medication trials in dementia.

## 2 METHODS

**FIGURE 1:**
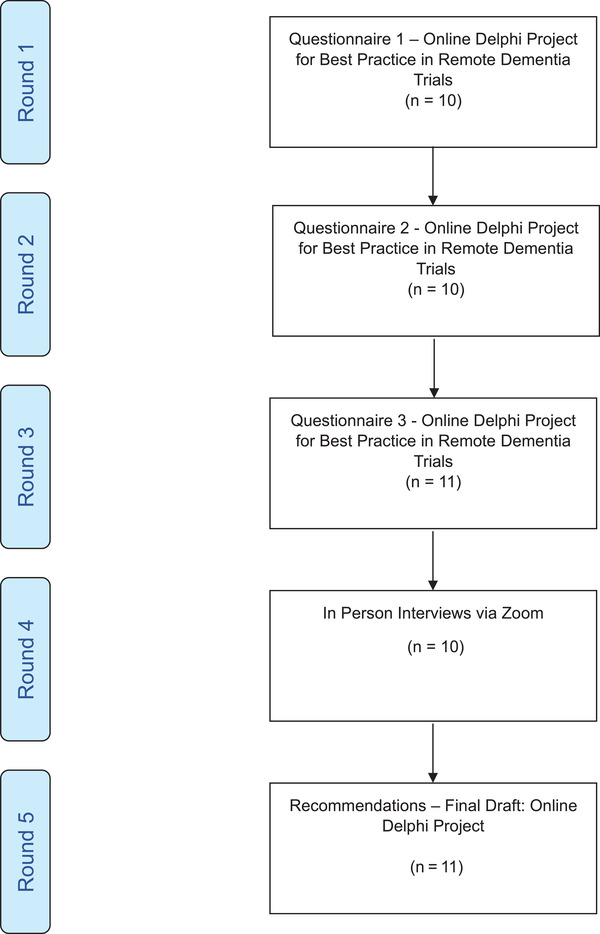
Flow chart showing the stages of the Delphi process

A five‐step modified Delphi process was employed based on prior successful work using such methodology^8^, ^9^ see Figure 1, Flowchart. The scope of the recommendations and initial statements were developed in consultation with members of the executive committee of the Alzheimer's Association International Society to Advance Alzheimer's Research and Treatment (ISTAART) Clinical Trials and Methodology Professional Interest Area (CTAM PIA) supplemented by two leading trialists in dementia with prior experience of leading successful Delphi processes in the field of dementia (P.K., G.J.).^9^, ^10^

The scope of the consensus process was limited to trials spanning phases 2b, 3, or 4, testing medications (investigational medicinal products) for the prevention of dementia. The type (etiology) of dementia was purposely not specified to allow adaptation spanning interventions that may include targets beyond Alzheimer's disease (AD) symptoms and or pathology. Dementia prevention was selected as a primary focus, that is, excluding trials in those who have diagnosed dementia but allowing inclusion of at risk populations including those who may be on the prodromal pathway. This was due to concerns that guidance for decentralized remote trials in those with a diagnosis of dementia would need to be considerably different and are, arguably, less practicable at the present time. An initial 24 statements were developed. These were under the headings of: Eligibility assessment, Trial medication, Safety (SAE/SUSAR) assessment, Data collection, Quality assurance, Retention, and Remote outcome assessment.

An international expert panel was engaged and asked to respond to the initial statements via an online questionnaire designed using Qualtrics software with anonymous responses collated by topic (e.g., safety reporting, outcome verification) and stored on secure University servers. Informed consent was obtained from all Delphi panelists prior to the start of engagement. Ethics approval was obtained from the University of New South Wales Human Research Ethics Committee (HREAP No.3678). Informed consent was obtained from all Delphi panelists.

Delphi panelists were asked to respond to each statement using a 6‐point Likert scale from strongly disagree, disagree, somewhat disagree, somewhat agree, agree, strongly agree, and were further invited to provide free text responses, comments or suggestions for further statements or nuances to guide subsequent rounds. Consensus was considered achieved if more than 70% of the expert group selected either agree/strongly agree or disagree/strongly disagree and broadly achieved if >70% selected either somewhat agree/agree/strongly agree, or the equivalent for disagree. To limit potential response bias, the Delphi panelists were blind to each other's identity and responses. The initial round of responses were collated by the research team and a second iteration was developed that incorporated free text comments to focus on areas where consensus was not achieved in the first round. This step was then repeated in one further round. Video‐conference discussions were then held with the expert panel members to more fully explore key areas where consensus was lacking or where responses were inconsistent. The discussions were led by the research team (R.P., L.H.) who presented the results and invited the panelist's views. These meetings were recorded, and the discussion used to inform the draft recommendations that were then circulated back to the panel with three options “agree,” “disagree,” “neutral.” The results were collated and finalized. The text of the recommendations was refined, with additional clarifications added, based on final comments from the panel members and the recommendations were recirculated to the panel for ratification. Because the infrastructure to support decentralized trials and the regulatory environment in which they operate varies by geographical region the finalized guidelines were then more widely circulated to the Alzheimer's Association ISTAART CTAM PIA. The CTAM PIA is an international grouping of over 1000 ISTAART members ranging from PhD students, early career researchers, regulators, and industry representatives to senior research academics with an interest in dementia and clinical trials. The CTAM PIA members were invited to join a virtual working group to respond to the final draft statements with the same three options “agree,” “disagree,” “neutral” and were provided with the opportunity to comment. PIA members were able to participate anonymously or to provide their details including their type of employer (university, government, industry, etc.) and prior experience with clinical trials. The responses from the PIA members were similarly collated with the percentage agreement and were compared to those of the expert panel. Areas lacking agreement were highlighted as a potential area for more in‐depth future work.

**RESEARCH IN CONTEXT**

1. **Systematic review**: The authors reviewed the literature using traditional methods. Whilst there is a growing body of evidence around the potential for using telehealth in clinical trials and the utility and validation of remote neuropsychological testing there remains a gap related to recommendations for the delivery of decentralized medication trials in the field of dementia.
2. **Interpretation**: The present work uses a modified Delphi process to develop a series of recommendations to support the design and delivery of decentralized medication trials for dementia prevention.
3. **Future directions**: We propose a framework for the development of trials in this area. Future work may expand our recommendations further to include dementia treatment trials.

## 3 RESULTS

Thirteen experts consented to be part of the panel bringing expertise in early and late phase trials, clinical, statistical, and subject area expertise and a range of career levels from early‐mid career to senior academic panel members. Panel members were based across the world in Europe, United Kingdom, Africa, North and South America, and Australia. Ten completed the first and second online rounds, 11 completed the third online round, and 10 were available for videoconference discussion (Figure 1).

In the first round, 24 statements were included in the initial questionnaire under the headings the Eligibility assessment, Trial medication, Safety (SAE/SUSAR) assessment, Data collection, Quality assurance, Retention and loss to follow‐up and Remote outcome assessment. For most statements, responses recording level of agreement were sought separately for trial phases 2b, 3, and 4. Additional questions were asked about the locations where study procedures could take place. Consensus was achieved for 47.5% of the statements (*n* = 32) (>70% selecting agree/strongly agree or disagree/strongly disagree) broadly achieved for a further 20% (*n* = 14) (>70% selecting somewhat agree/agree/strongly agree or somewhat disagree/disagree/strongly disagree) but not for the remaining 32% (*n* = 22). Areas that failed to achieve consensus in the first Delphi round included data collection methods, quality assurance and adherence, the need for in person dispensing and in person outcome assessment. There was agreement around the level of remoteness for the locations where trial tasks could take place (Table 1).

**TABLE 1:** Consensus on acceptable remoteness for trial procedures

Procedures that can take place:		Percentage agreement
At a participant's home	Blood draw	88%
	Electrocardiogram (ECG)	75%
	Fitting of wearables	75%
	Collection of wearables	88%
At a healthcare provider close to participants home	Blood draw ECG	88% 88%
At a Trial site	Blood draw	75%
	ECG	75%
	Electroencephalogram (EEG)	75%
	Imaging	100%
	Cerebrospinal fluid (CSF) sampling	88%
	Fitting of wearables	100%
	Collection of wearables	75%
Online	Interview based tools to assess dementia severity or change	88%
	Interview tools to assess mood	100%

The second questionnaire included 43 statements on the areas that did not achieve consensus in the prior round and, informed by free text comments from participants, resulted in strong consensus achieved for 28% (*n* = 12), broad consensus for 56% (*n* = 24), and none for 16% (*n* = 7).

The third round included 19 statements that further refined the areas of disagreement. For the third round 37% (*n* = 7) achieved strong consensus, 26% (*n* = 5) achieved broad consensus and 37% (*n* = 7) were without consensus. The remaining areas lacking consensus focused on assessing adherence to trial medication, physician assessment after report of a serious adverse event (SAE), use of medical records for outcome data and in person dispensing.

Following the third questionnaire, discussion at two expert videoconference panel meetings and three one‐to‐one meetings resulted in agreed final draft recommendations with 40 statements with three responses “Agree, Neutral, Disagree.” Eleven panel members completed the final questionnaire and reached consensus for all 40 statements (Supplementary Text A) with additional clarification added to five statements based on final free‐text comments from the expert panel.

Figure 2 shows the final recommendations from the expert panel. Overall, the recommendations show the delivery of decentralized trials in dementia prevention to be feasible with adequate safety checks and balances.

FIGURE 2Recommendations for decentralized trials of medicinal products for dementia prevention, phases 2b, 3, and 4

**Figure:**
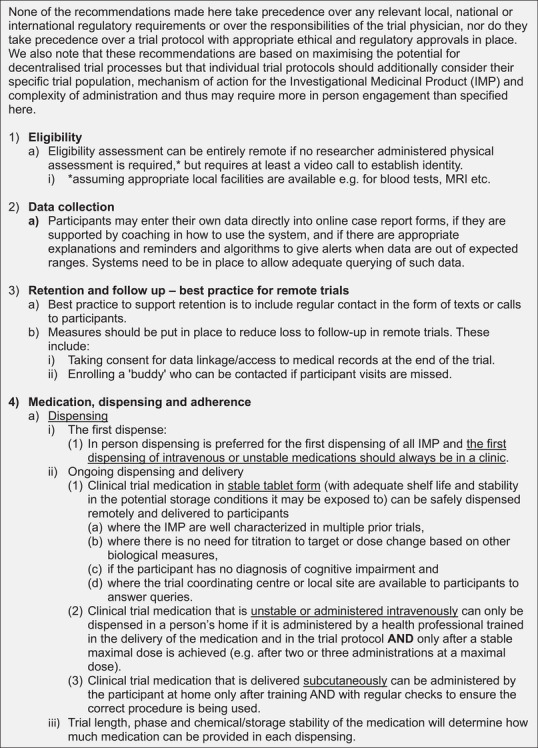

**Figure:**
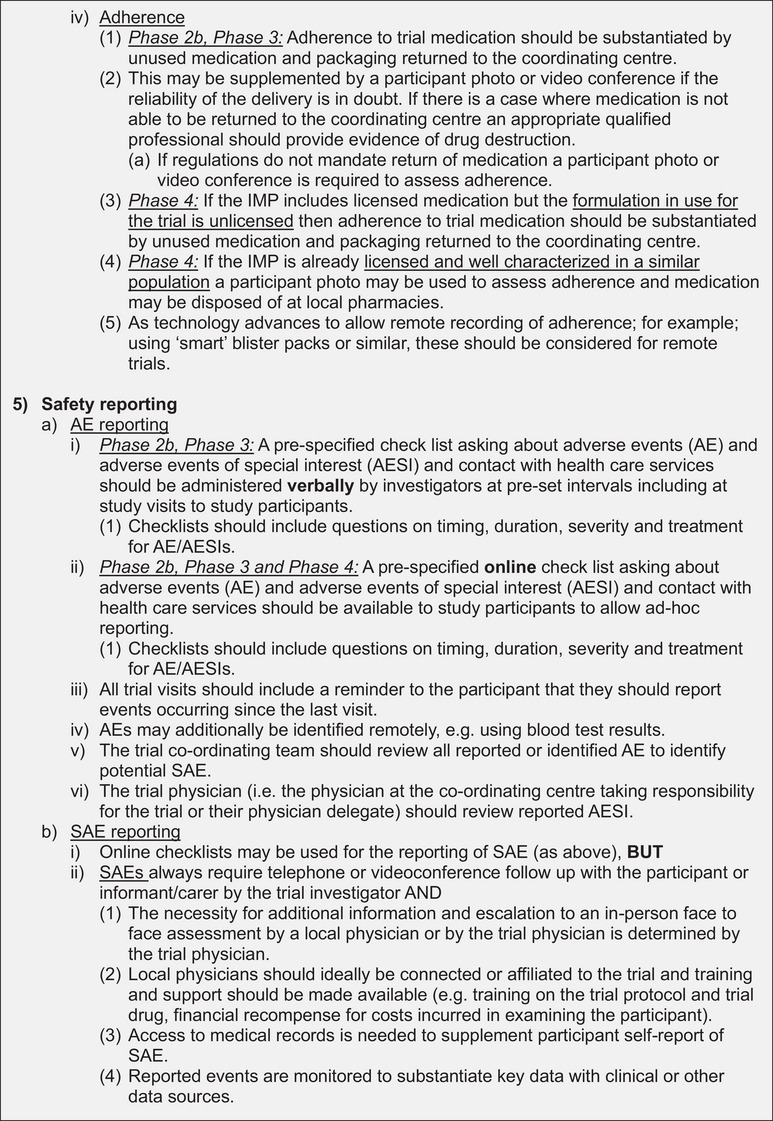

**Figure:**
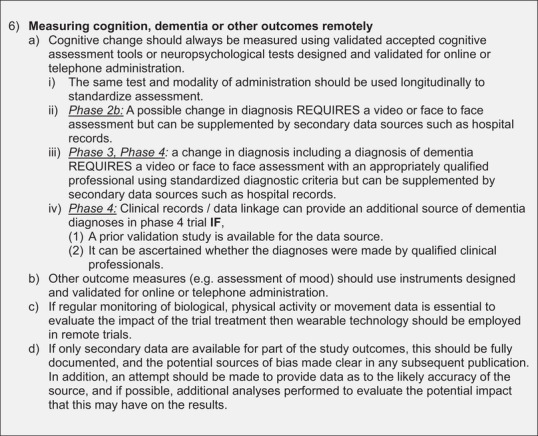

### 3.1 Validation and comment by the CTAM PIA

Twenty members of the PIA provided informed consent to participate in the working group review of the recommendations. Seventeen provided responses, one of whom elected to remain anonymous. One person completed the questionnaire twice with 73% agreement between their attempts, their most recent completion was used for analysis. Almost 90% (*n* = 15) reported their primary employer was university or not for profit research institute, one person (6%) was employed in the pharmaceutical industry, and one (6%) by government. Four (24%) identified primarily as clinicians with the rest (76%) describing themselves as researchers. Experience with medication trials in dementia risk reduction ranged from 0 to 5 years (47%, *n* = 8), 6 to 10 years (24%, *n* = 4), 11 to 15 years (6% *n* = 1), and four (24%) reported having more than 20 years experience. Twelve PIA members completed all questions, and in general, the PIA members showed agreement with the expert committee, with the majority (>50%) of the PIA respondents selecting “agree” for all 40 consensus statements (Supplementary Text B). There were no statements that the PIA members disagreed with. Strong agreement (>70%) was obtained for 29 statements; this included total agreement (100% selecting “agree”) for 8 statements and either no one selecting “disagree” (*n* = 14 statements) or just one person selecting “disagree” (*n* = 6 statements). General agreement was obtained for a further 11 statements with the level of agreement between 50% and 70%, but where the “agree” and “neutral” categories taken together accounted for >70% of responses meaning the level of disagreement was low.

There were two consensus statements where the responses were most mixed, both related to trial medication. For the first, the statement “Clinical trial medication in stable tablet form (with adequate shelf life and stability in the potential storage conditions it may be exposed to) can be safely dispensed remotely and delivered to participants,” 53% of the PIA group responding to this statement agreed (*n* = 10), 46% were neutral, and no‐one disagreed. Similarly, for the subsequent statement “Clinical trial medication that is unstable or administered intravenously can only be dispensed in a person's home if it is administered by a health professional trained in the delivery of the medication and in the trial protocol AND only after a stable maximal dose is achieved (e.g., after two or three administrations at a maximal dose) 53% of the PIA group responding to this statement agreed (*n* = 10), 20% were neutral, however, four people disagreed.” There were no free text comments that provided additional insight into the responses to these two statements. The comments that were received from this group highlighted additional areas to evaluate, including the potential for validating additional tools for remote use, and the applicability of remote trials to specific population groups including the potential preference of participants from some population groups for face‐to‐face contact.

## 4 DISCUSSION

We provide 40 consensus‐based recommendations for the conduct of remote clinical medication trials from phase 2b onward to phase IV in dementia prevention. Our recommendations are designed to complement the emerging general guidelines by focusing specifically on dementia and using expert opinions in the dementia field. Specifically, our recommendations are to aid researchers in the design and delivery of remote trials. Our recommendations are not to replace or dictate particular requirements for any given trial, as specifics are necessarily governed by the protocol approved by an appropriate human research ethics committee, and must be in adherence to the applicable regulatory environment.

Strengths of our study include the composition and commitment of our Delphi panel members who brought expertise and experience across the dementia medication trial and dementia risk reduction field representing clinical trial researchers at all career levels and with a breadth of dementia‐related backgrounds in physiology, medicine (neurology psychiatry), pharmacology, and psychology. Whereas it was not possible to have all panel members on the same teleconference discussion due to time differences and availability, this did mean that panel members were based globally and able to contribute international perspectives, specifically from Greece, the United Kingdom, Nigeria, the United States of America, Argentina, and Australia. Furthermore, although the representativeness of the panel was inevitably limited due to its size, the recommendations were further ratified and commented on by members of the CTAM PIA working group for remote trials representing the United Kingdom, United States of America, Netherlands, Greece, Chile, Brazil, and India.

In general, there was broad consensus around the key aspects of decentralized trials and although some statements may seem obvious to some readers, our intent was to produce recommendations that are accessible to a broad range of research disciplines with different levels of expertise and to provide a benchmark in the field. Going forward, additional discussion may be required around the specifics of remote dispensing, delivery, and disposal as these may be influenced by local infrastructure and facilities and showed the lowest level of ratification from the PIA members. In particular, when involving home administration of intravenous medication once a stable dose has been achieved in a clinical setting. It should also be noted that although the levels of agreement are lower among our PIA members, they responded online, whereas the expert panel was able to engage in discussion, which potentially facilitated understanding. It is important to reiterate that our recommendations are not to replace or dictate particular specifications for any given trial. Trial specifics are necessarily governed by the protocol approved by an appropriate human research ethics committee and must be in adherence to the applicable regulatory environment. For example, some trials may need to maintain detailed communication pathways with local emergency or healthcare providers and appropriate trial training should always be provided.

## 5 CONCLUSION

Working with an expert review panel and an international trials working group we used Delphi methodology to produce an accessible and practical guide for the design and conduct of remote medication trials in dementia prevention.

## CONFLICT OF INTEREST STATEMENT

Leanne Howard reports honoraria for guest lectures from the University on New South Wales. Carla Abdelnour has received a postdoctoral fellowship from the Susan and Charles Berghoff Foundation. Leadership roles in the last 36 months include ISTAART Clinical Trials and Methodology Professional Interest Area Professional Interest Area Program Chair and Early‐Career Representative (unpaid). In the last 36 months, she has received honoraria as speaker from F. Hoffmann‐La Roche Ltd, Nutricia, Schwabe Farma Iberica S.A.U. She is a member of the Board of Directors of the Lewy Body Dementia Association and coordinator of the cognitive and behavioural studies group of te Catalan Society of Neurology. Erin Abner has received grants paid to her institution from the NIH. Leadership roles in the last 36 months include ISTAART Clinical Trials and Methodology Professional Interest Area Professional Interest Area Chair and Vice‐Chair (unpaid). Honoraria from IMPACT‐AD as program faculty and from ACTC Recruitment, Education and Retention Unit as External Asvisory Board Member. Ricardo F. Allegri has received grants paid to his institution from CONICET, Fleni, and the Alzheimer's Association. Hiroko H. Dodge has received grants paid to her institution from the NIH R01AG051628 R01AG056102 R01AG056712 P3OAG008017 R01AG042191 PO1AG043362 RO1AG043398 RO1AG058687 R01AG042191 P30AG008017 P30AG024978 U2CAG054397 R01AG056712 R01AG038651 P30AG053760 U01NS100611 U2C AG057441 U01NS106670 R01AG054484 over the last 36 months. She has participated on data safety monitoring boards/advisory boards for which she has received honoraria. She has recieved consulting fees from Northwestern ADC, Florida 1 ADC, Centers of Biomedical Research Excellence at University of Hawaii, honoraria from the Impact‐AD workshop supported by ACTC in the last 36 months. Leadership roles in the last 36 months include membership of the ISTAART Advisory Board. Serge Gauthier reports membership of scientific advisory boards of Alzheon, AmyriAD, Biogen, Eisai, Enigma USA, Lilly, Lundbeck, NovoNordisk, Okutsa, TauRx. Leadership roles for the Sharon Francis Foundation Toronto, he is on editorial boards for Neurotoriun, JPAD Camilla Hoyos, reports no conflict of interest. Gregory A. Jicha has received grants paid to his institution from NIH P30AG072946 R01AG075959. Patrick G Kehoe, reports membership of a DSMB for a decentralised feasibility trial. Catherine Mummery, has received grants paid to her institution from Biogen over the last 36 months. She has received consulting fees and honoraria from Eli Lilly; she has sat on advisory boards or held leadership roles for Biogen, Eisai, Roche, Eli Lilly, Novartis, and has participated on data safety monitoring committees in the last 36 months. Adesola Ogunniyi, reports no conflict of interest. Nikolaos Scarmeas, has received grants paid to his institution in his role as a local PI of recruiting site for a multinational, multicenter NovoNordisk sponsored phase III treatment trial for Alzheimer's disease and grants paid to him from Albert Einstein College of Medicine in his role as Chair of Data Safety Monitoring Board of an NIH funded study. Xiaoying Chen reports no conflict of interest. Jodie Titiner, is an employee of the Alzheimer's Association and reports no conflict of interest Christopher Weber is an employee of the Alzheimer' Association and reports no conflict of interest. Ruth Peters has received grants paid to her institution from the Australian NHMRC and the University of New South Wales in the past 36 months. Leadership roles in the last 36 months include Chair of the Alzheimer's Association International Society to Advance Alzheimer's Research and Treatment (ISTAART) Clinical Trials and Methodology Professional Interest Area (unpaid). Author disclosures are available in the supporting information.

## CONSENT STATEMENT

All human participants provided informed consent.

## Supporting information

Supporting Information

Supporting Information
