# Supplementary material for: Decentralized clinical trials for medications to reduce the risk of dementia: Consensus report and guidance
Source: Alzheimers Dement. 2024 Jun 2;20(7):4625–34. doi: 10.1002/alz.13891 (PMC11247660; doi:10.1002/alz.13891)
Supplement: Supplementary file 1 — Supporting Information [file ALZ-20-4625-s002.docx]

Supplementary files

Table A: Final recommendations showing percentage agreement from the expert committee.

| **Eligibility** | **Agree** | **Neutral** | **Disagree** |
| --- | --- | --- | --- |
| 1: Eligibility assessment can be entirely remote if no researcher administered physical assessment is required,* but requires at least a video call to establish identity.  *assuming appropriate local facilities are available e.g. for blood tests, MRI etc. | 90.9% | 0.0% | 9.1% |
| **Data Collection** | **Agree** | **Neutral** | **Disagree** |
| 2: Participants may enter their own data directly into online case report forms, if they are supported by coaching in how to use the system, and if there are appropriate explanations and reminders and algorithms to give alerts when data are out of expected ranges. Systems need to be in place to allow adequate querying of such data. | 100% | 0% | 0% |
| **Retention and follow up - best practice for remote trials:** | **Agree** | **Neutral** | **Disagree** |
| 3: Best practice to support retention is to include regular contact in the form of texts or calls to participants. | 90.9% | 9.1% | 0.0% |
| 4: Measures should be put in place to reduce loss to follow-up in remote trials. | 100.0% | 0.0% | 0.0% |
| These include: |  |  |  |
| 5: Taking consent for data linkage/access to medical records at the end of the trial. | 90.9% | 0.0% | 9.1% |
| 6: Enrolling a ‘buddy’ who can be contacted if participant visits are missed. | 100.0% | 0.0% | 0.0% |
| **Medication, dispensing and adherence.** | **Agree** | **Neutral** | **Disagree** |
| *Dispensing* | | | |
| *The first dispense:* | | | |
| 7: In person dispensing is preferred for the first dispensing of all IMP and the first dispensing of intravenous or unstable medications should always be in a clinic. | 100.0% | 0.0% | 0.0% |
| *Ongoing dispensing and delivery* | | | |
| 8: Clinical trial medication in stable tablet form (with adequate shelf life and stability in the potential storage conditions it may be exposed to) can be safely dispensed remotely and delivered to participants   - where the IMP are well characterized in multiple prior trials, - where there is no need for titration to target or dose change based on other biological measures, - if the participant has no diagnosis of cognitive impairment and - where the trial coordinating centre or local site are available to participants to answer queries. | 100.0% | 0.0% | 0.0% |
| 9: Clinical trial medication that is unstable or administered intravenously can only be dispensed in a person’s home if it is administered by a health professional trained in the delivery of the medication and in the trial protocol AND only after a stable maximal dose is achieved (e.g. after two or three administrations at a maximal dose). | 100.0% | 0.0% | 0.0% |
| 10: Clinical trial medication that is delivered subcutaneously can be administered by the participant at home only after training AND with regular checks to ensure the correct procedure is being used | 100.0% | 0.0% | 0.0% |
| 11: Trial length, phase and chemical/storage stability of the medication will determine how much medication can be provided in each dispensing. | 100.0% | 0.0% | 0.0% |
| *Adherence* | | | |
| 12: Phase 2b, Phase 3: Adherence to trial medication should be substantiated by unused medication and packaging returned to the coordinating centre. | 90.9% | 9.1% | 0.0% |
| 13: This may be supplemented by a participant photo or video conference if the reliability of the delivery is in doubt. | 81.8% | 9.1% | 9.1% |
| 14: If there is a case where medication is not able to be returned to the coordinating centre an appropriate qualified professional should provide evidence of drug destruction. | 81.8% | 9.1% | 9.1% |
| 15: If regulations do not mandate return of medication a participant photo or video conference is required to assess adherence. | 81.8% | 9.1% | 9.1% |
| 16: Phase 4: If the IMP includes licensed medication but the formulation in use for the trial is unlicensed then adherence to trial medication should be substantiated by unused medication and packaging returned to the coordinating centre. | 81.8% | 18.2% | 0.0% |
| 17: Phase 4: If the IMP is already licensed and well characterized in a similar population a participant photo may be used to assess adherence and medication may be disposed of at local pharmacies. | 90.9% | 9.1% | 0.0% |
| 18: As technology advances to allow remote recording of adherence; for example; using ‘smart’ blister packs or similar, these should be considered for remote trials. | 100.0% | 0.0% | 0.0% |
| **Safety reporting** | **Agree** | **Neutral** | **Disagree** |
| *AE reporting* | | | |
| 19: Phase 2b, Phase 3: A pre-specified check list asking about adverse events (AE) and adverse events of special interest (AESI) and contact with health care services should be administered verbally by investigators at pre-set intervals, including at study visits to study participants | 72.7% | 18.2% | 9.1% |
| 20: Checklists should include questions on timing, duration, severity and treatment for AE/AESIs. | 100.0% | 0.0% | 0.0% |
| 21: Phase 2b, Phase 3 and Phase 4: A pre-specified online check list asking about adverse events (AE) and adverse events of special interest (AESI) and contact with health care services should be available to study participants to allow ad-hoc reporting. | 90.9% | 0.0% | 9.1% |
| 22: Checklists should include questions on timing, duration, severity and treatment for AE/AESIs. | 100.0% | 0.0% | 0.0% |
| 23: All trial visits should include a reminder to the participant that they should report events occurring since the last visit. | 100.0% | 0.0% | 0.0% |
| 24: AEs may additionally be identified remotely, e.g. using blood test results. | 90.9% | 0.0% | 9.1% |
| 25: The trial co-ordinating team should review all reported or identified AE to identify potential SAE | 90.9% | 9.1% | 0.0% |
| 26: The trial physician (i.e. the physician at the co-ordinating centre taking responsibility for the trial or their physician delegate) should review reported AESI. | 100.0% | 0.0% | 0.0% |
| *SAE reporting* | | | |
| 27: Online checklists may be used for the reporting of SAE (as above) BUT SAEs always require telephone or videoconference follow up with the participant or informant/carer by the trial investigator | 90.9% | 0.0% | 9.1% |
| 28: The necessity for additional information and escalation to an in-person face to face assessment by a local physician or by the trial physician is determined by the trial physician. | 100.0% | 0.0% | 0.0% |
| 29: Local physicians should ideally be connected or affiliated to the trial and training and support should be made available (e.g. training on the trial protocol and trial drug, financial recompense for costs incurred in examining the participant). | 90.9% | 9.1% | 0.0% |
| 30: Access to medical records is needed to supplement participant self-report of SAE | 100.0% | 0.0% | 0.0% |
| 31: Reported events are monitored to substantiate key data with clinical or other data sources. | 100.0% | 0.0% | 0.0% |
| **Measuring cognition, dementia or other outcomes** | **Agree** | **Neutral** | **Disagree** |
| 32: Cognitive change should always be measured using validated accepted cognitive assessment tools or neuropsychological tests designed and validated for online or telephone administration. | 90.9% | 0.0% | 9.1% |
| 33: The same test and modality of administration should be used longitudinally to standardize assessment. | 100.0% | 0.0% | 0.0% |
| 34: Phase 2b: A possible change in diagnosis REQUIRES a video or face to face assessment but can be supplemented by secondary data sources such as hospital records. | 90.9% | 9.1% | 0.0% |
| 35: Phase 3, Phase 4: a change in diagnosis including a diagnosis of dementia REQUIRES a video or face to face assessment with an appropriately qualified professional using standardized diagnostic criteria but can be supplemented by secondary data sources such as hospital records. | 100.0% | 0.0% | 0.0% |
| Phase 4: Clinical records / data linkage can provide an additional source of dementia diagnoses in phase 4 if; | | | |
| 36: A prior validation study is available for the data source | 100.0% | 0.0% | 0.0% |
| 37: It can be ascertained whether the diagnoses were made by qualified clinical professionals | 100.0% | 0.0% | 0.0% |
| 38: Other outcome measures (e.g. assessment of mood) should use instruments designed and validated for online or telephone administration. | 100.0% | 0.0% | 0.0% |
| 39: If regular monitoring of biological, physical activity or movement data is essential to evaluate the impact of the trial treatment then wearable technology should be employed in remote trials. | 90.9% | 9.1% | 0.0% |
| 40: If only secondary data are available for part of the study outcomes this should be fully documented, and the potential sources of bias made clear in any subsequent publication. In addition, an attempt should be made to provide data as to the likely accuracy of the source, and if possible, additional analyses performed to evaluate the potential impact that this may have on the results. | 100.0% | 0.0% | 0.0% |

|  |  |  |  |  |  |
| --- | --- | --- | --- | --- | --- |

Rounding may mean that values do not add up to 100%

Table B: Final recommendations showing percentage agreement from the PIA Members.

| **Eligibility** | **Agree** | **Neutral** | **Disagree** |
| --- | --- | --- | --- |
| 1: Eligibility assessment can be entirely remote if no researcher administered physical assessment is required,* but requires at least a video call to establish identity.  *assuming appropriate local facilities are available e.g. for blood tests, MRI etc. | 82% | 6% | 12% |
| **Data Collection** | **Agree** | **Neutral** | **Disagree** |
| 2: Participants may enter their own data directly into online case report forms, if they are supported by coaching in how to use the system, and if there are appropriate explanations and reminders and algorithms to give alerts when data are out of expected ranges. Systems need to be in place to allow adequate querying of such data. | 59% | 29% | 12% |
| **Retention and follow up - best practice for remote trials:** | **Agree** | **Neutral** | **Disagree** |
| 3: Best practice to support retention is to include regular contact in the form of texts or calls to participants. | 76% | 24% | 0% |
| 4: Measures should be put in place to reduce loss to follow-up in remote trials. | 100.0% | 0.0% | 0.0% |
| These include: |  |  |  |
| 5: Taking consent for data linkage/access to medical records at the end of the trial. | 59% | 35% | 6% |
| 6: Enrolling a 'buddy' who can be contacted if participant visits are missed. | 65% | 29% | 6% |
| **Medication, dispensing and adherence.** | **Agree** | **Neutral** | **Disagree** |
| *Dispensing* | | | |
| *The first dispense:* | | | |
| 7: In person dispensing is preferred for the first dispensing of all IMP and the first dispensing of intravenous or unstable medications should always be in a clinic. | 81% | 13% | 6% |
| *Ongoing dispensing and delivery* | | | |
| 8: Clinical trial medication in stable tablet form (with adequate shelf life and stability in the potential storage conditions it may be exposed to) can be safely dispensed remotely and delivered to participants  • where the IMP are well characterized in multiple prior trials,  • where there is no need for titration to target or dose change based on other biological measures,  • if the participant has no diagnosis of cognitive impairment and  • where the trial coordinating centre or local site are available to participants to answer queries. | 53% | 47% | 6% |
| 9: Clinical trial medication that is unstable or administered intravenously can only be dispensed in a person’s home if it is administered by a health professional trained in the delivery of the medication and in the trial protocol AND only after a stable maximal dose is achieved (e.g. after two or three administrations at a maximal dose). | 53% | 20% | 27% |
| 10: Clinical trial medication that is delivered subcutaneously can be administered by the participant at home only after training AND with regular checks to ensure the correct procedure is being used | 67% | 20% | 13% |
| 11: Trial length and phase and chemical/storage stability of the medication will determine how much medication can be provided in each dispensing. | 67% | 33% | 0% |
| *Adherence* | | | |
| 12: Phase 2b, Phase 3: Adherence to trial medication should be substantiated by unused medication and packaging returned to the coordinating centre. | 93% | 7% | 0% |
| 13: This may be supplemented by a participant photo or video conference if the reliability of the delivery is in doubt. | 79% | 21% | 0% |
| 14: If there is a case where medication is not able to be returned to the coordinating centre an appropriate qualified professional should provide evidence of drug destruction. | 93% | 0% | 7% |
| 15: If regulations do not mandate return of medication a participant photo or video conference is required to assess adherence. | 79% | 21% | 0% |
| 16: Phase 4: If the IMP includes licensed medication but the formulation in use for the trial is unlicensed then adherence to trial medication should be substantiated by unused medication and packaging returned to the coordinating centre. | 64% | 36% | 0% |
| 17: Phase 4: If the IMP is already licensed and well characterized in a similar population a participant photo may be used to assess adherence and medication may be disposed of at local pharmacies. | 57% | 29% | 14% |
| 18: As technology advances to allow remote recording of adherence; for example; using ‘smart’ blister packs or similar, these should be considered for remote trials. | 100.0% | 0.0% | 0.0% |
| **Safety reporting** | **Agree** | **Neutral** | **Disagree** |
| *AE reporting* | | | |
| 19: Phase 2b, Phase 3: A pre-specified check list asking about adverse events (AE) and adverse events of special interest (AESI) and contact with health care services should be administered verbally by investigators at pre-set intervals, including at study visits to study participants | 100% | 0% | 0% |
| 20: Checklists should include questions on timing, duration, severity and treatment for AE/AESIs. | 100.0% | 0.0% | 0.0% |
| 21: Phase 2b, Phase 3 and Phase 4: A pre-specified online check list asking about adverse events (AE) and adverse events of special interest (AESI) and contact with health care services should be available to study participants to allow ad-hoc reporting. | 57% | 29% | 14% |
| 22: Checklists should include questions on timing, duration, severity and treatment for AE/AESIs. | 71% | 21% | 7% |
| 23: All trial visits should include a reminder to the participant that they should report events occurring since the last visit. | 100.0% | 0.0% | 0.0% |
| 24: AEs may additionally be identified remotely, e.g. using blood test results. | 93% | 7% | 0% |
| 25: The trial co-ordinating team should review all reported or identified AE to identify potential SAE | 100.0% | 0.0% | 0.0% |
| 26: The trial physician (i.e. the physician at the co-ordinating centre taking responsibility for the trial or their physician delegate) should review reported AESI. | 100.0% | 0.0% | 0.0% |
| *SAE reporting* | | | |
| 27: Online checklists may be used for the reporting of SAE (as above) BUT SAEs always require telephone or videoconference follow up with the participant or informant/carer by the trial investigator | 92% | 0% | 8% |
| 28: The necessity for additional information and escalation to an in-person face to face assessment by a local physician or by the trial physician is determined by the trial physician. | 75% | 25% | 0% |
| 29: Local physicians should ideally be connected or affiliated to the trial and training and support should be made available (e.g. training on the trial protocol and trial drug, financial recompense for costs incurred in examining the participant). | 92% | 8% | 0% |
| 30: Access to medical records is needed to supplement participant self-report of SAE | 75% | 17% | 8% |
| 31: Reported events are monitored to substantiate key data with clinical or other data sources. | 83% | 17% | 0% |
| **Measuring cognition, dementia or other outcomes** | **Agree** | **Neutral** | **Disagree** |
| 32: Cognitive change should always be measured using validated accepted cognitive assessment tools or neuropsychological tests designed and validated for online or telephone administration. | 92% | 8% | 0% |
| 33: The same test and modality of administration should be used longitudinally to standardize assessment. | 100.0% | 0.0% | 0.0% |
| 34: Phase 2b: A possible change in diagnosis REQUIRES a video or face to face assessment but can be supplemented by secondary data sources such as hospital records. | 92% | 8% | 7.5% |
| 35: Phase 3, Phase 4: a change in diagnosis including a diagnosis of dementia REQUIRES a video or face to face assessment with an appropriately qualified professional using standardized diagnostic criteria but can be supplemented by secondary data sources such as hospital records. | 92% | 8% | 0% |
| Phase 4: Clinical records / data linkage can provide an additional source of dementia diagnoses in phase 4 if; | | | |
| 36: A prior validation study is available for the data source | 67% | 25% | 8% |
| 37: It can be ascertained whether the diagnoses were made by qualified clinical professionals | 83% | 17% | 0% |
| 38: Other outcome measures (e.g. assessment of mood) should use instruments designed and validated for online or telephone administration. | 92% | 8% | 0% |
| 39: If regular monitoring of biological, physical activity or movement data is essential to evaluate the impact of the trial treatment then wearable technology should be employed in remote trials. | 83% | 8% | 8% |
| 40: If only secondary data are available for part of the study outcomes this should be fully documented, and the potential sources of bias made clear in any subsequent publication. In addition, an attempt should be made to provide data as to the likely accuracy of the source, and if possible, additional analyses performed to evaluate the potential impact that this may have on the results. | 92% | 8% | 0% |

Rounding may mean that values do not add up to 100%
